# 非小细胞肺癌吉西他滨药物耐药相关基因研究进展

**DOI:** 10.3779/j.issn.1009-3419.2011.05.08

**Published:** 2011-05-20

**Authors:** 莹 陈, 晓萍 钱, 宝瑞 刘

**Affiliations:** 1 210008 南京，南京医科大学鼓楼临床医学院肿瘤中心 Cancer Center, Drum Tower Medical School, Nanjing Medical University, Nanjing 210008, China; 2 210008 南京，南京大学医学院附属鼓楼医院肿瘤中心 Cancer Center, Afliated Drum Tower Hospital, Medical School of Nanjing University, Nanjing 210008, China

**Keywords:** 肺肿瘤, 吉西他滨, 平衡型核苷转运载体1, 核糖核苷酸还原酶亚基1, 脱氧胞苷激酶, Lung neoplasms, Gemcitabine, Human equilibrative nucleoside transporter1, Ribonucleotide reductase M, Deoxicytidine kinase

## Abstract

随着药物基因组学、药物遗传学的发展，基因指导下个体化治疗成为提高非小细胞肺癌（non-small cell lung cancer, NSCLC）化疗疗效的有效途径之一。确定药物的相关预测性分子标志物从而指导临床治疗、提高疗效被广泛关注。吉西他滨是目前NSCLC常用化疗药物之一，本文主要阐述了近年来吉西他滨药物耐药相关基因在NSCLC个体化治疗方面的研究进展。

## Abstract (en)

With the development of pharmacogenomics and pharmacogenetics, personal therapy based on genes has become one of the most effective ways to enhance chemotherapeutic effect on non-small cell lung cancer (NSCLC) patients. Much attention has been paid to validate the predictive biomarkers of chemotherapy in order to guide chemotherapy and enhance effect in general. Gemcitabine is one of the common agents treating NSCLC recently. This review is mainly about the recent reports on potential biomarkers of Gemcitabine in tailored therapy of NSCLC.

吉西他滨是目前非小细胞肺癌（non-small cell lung cancer, NSCLC）常用的化疗药物之一，其在NSCLC的一线治疗中已取得较好的疗效。然而由于不同个体间肿瘤细胞的生物学特性差异较大，对化疗药物的敏感性差异亦大，使其在单药治疗初治或既往化疗失败患者的总有效率仅为20%左右^[1]^。随着药物基因组学、药物遗传学的发展，基因指导下个体化治疗成为提高NSCLC化疗疗效的有效途径之一，确定药物的相关预测性分子标志物从而指导临床治疗、提高疗效被广泛关注。现就近年来吉西他滨药物耐药相关基因在NSCLC个体化治疗方面的研究进展作一综述。

吉西他滨是嘧啶类抗代谢药物，属于细胞周期特异性药物，主要作用于DNA合成期，在一定条件下也可以阻止细胞从合成前期向合成期进展。吉西他滨进入肿瘤细胞内被转化为具有活性的吉西他滨磷酸盐，影响DNA合成和修复，从而抑制肿瘤细胞分裂，诱导肿瘤细胞凋亡。肿瘤细胞对吉西他滨的药物抵抗主要涉及药物转运、药物代谢、药物作用靶点这三个方面。

## 1 药物转运相关基因

### 1.1 平衡型核苷转运载体1（human equilibrative nucleoside transporter1, hENT1）

吉西他滨为亲水性物质，不能通过自由扩散方式进入细胞内，而需要相应的载体如hENT1、hENT2、聚集型核苷转运载体（human concentrate nucleoside transporter）1、2、3（hCNT1、hCNT2、hCNT3）转运入细胞内，其中起主要作用的是hENT1，缺乏hENT1的细胞对核苷类似物有高度抵抗作用^[2]^。日本名古屋大学医学研究所Achiwa等^[3]^通过RT-PCR方法检测了22种NSCLC细胞株的hENT1 mRNA表达水平，来研究其与吉西他滨敏感性的关系，结果显示hENT1 mRNA表达水平与吉西他滨半数抑制浓度（50% inhibiting concentration, IC_50_）呈负相关（*r*=-0.676, 9, *P*=0.000, 5)，因此得出结论hENT1 mRNA上调可以增加吉西他滨药物对NSCLC细胞株敏感性，hENT1可以作为吉西他滨治疗NSCLC的预测分子。该研究小组同时通过Western blot方法检测了14种NSCLC细胞的hENT1蛋白表达，并研究其与*hENT1*基因表达以及吉西他滨药物敏感性的关系^[4]^，结果显示hENT1蛋白表达与其基因表达明显相关（*r*=0.533, *P* < 0.05)，并且其蛋白表达与吉西他滨药物IC_50_呈负相关（*r*=-0.622, *P* < 0.05），并采用免疫组化方法检测了接受吉西他滨化疗的24例NSCLC患者石蜡组织切片的hENT1蛋白表达，结果显示hENT1蛋白表达阳性患者在含吉西他滨治疗中明显获益（*P* < 0.05），与体外研究结果一致。意大利Toffalorio1等^[5]^用siRNA方法下调了两株肺癌细胞株A549、H1703的hENT1 mRNA表达水平后发现，吉西他滨IC_50_值明显提高（*P*=0.018），可以得出与上同样的结论。而国内关于hENT1在NSCLC吉西他滨药物敏感性方面的研究则罕见报道。在*hENT1*的基因多态性方面，美国宾西法尼亚洲的彼兹堡大学Myers等^[6]^研究发现携带hENT1 CGG/CGC基因型者hENT1 mRNA表达水平较高（*P*=0.12），由此可以推论*hENT1*的基因多态性可能通过影响hENT1 mRNA表达水平从而影响吉西他滨药物敏感性。

### 1.2 ATP结合小盒（ATP-binding cassette, ABC）转运载体

ABC转运载体可以将细胞内的药物泵出细胞外，与核苷转运载体共同维持细胞内药物浓度的平衡。这类基因或者表达的蛋白参与多药耐药（multidrug resistance, MDR），主要包括p-葡萄糖蛋白（p-glyco-protein, p-gp）和MRP5（ABCC5）。荷兰VU大学医学中心Bergman等^[7]^研究了人体包括NSCLC在内的五种不同肿瘤细胞株及其p-gp、MRP1（ABCC1）过表达的变异细胞株与吉西他滨药物敏感性关系以及多药耐药细胞对吉西他滨敏感性的机制，结果显示，NSCLC的4种变异细胞与对照组相比均对吉西他滨敏感（*P* < 0.01），表明p-gp、MRP1过表达细胞对吉西他滨更加敏感，其可能机制为：多药耐药引起的脱氧胞苷激酶（deoxicytidine kinase, dck）表达增高进一步增加了细胞内的吉西他滨三磷酸盐（dFdCTP）积累，并且促进吉西他滨结合到肿瘤细胞DNA链从而终止其延长，使得其对吉西他滨更加敏感。因此，多药耐药的肿瘤复发患者，可以选择检测p-gp、MRP1表达水平作为吉西他滨临床治疗的预测指标。日本Oguri等^[8]^通过RT-PCR方法检测了17种NSCLC细胞株的ABCC5 mRNA表达水平，分析其与吉西他滨敏感性的关系，结果显示ABCC5 mRNA表达水平与吉西他滨敏感性呈负相关（*P* < 0.01），并且使用ABCC5抑制剂扎普斯特分别作用于两种NSCLC细胞株后，其对吉西他滨药物敏感性明显增加；同时采用siRNA方法作用于细胞株，细胞株ABCC5 mRNA的表达水平明显降低，吉西他滨药物敏感性明显增加。但是目前关于ABC转运载体相关基因仍主要集中于体外研究，此类基因是否可以作为NSCLC吉西他滨药物敏感性预测相关基因尚需要大量大样本临床回顾性及多中心前瞻性临床研究。

## 2 药物代谢相关基因

### 2.1 脱氧胞苷激酶（deoxicytidine kinase, dck）

吉西他滨进入细胞后，首先在细胞内通过脱氧胞苷激酶的作用被磷酸化为单磷酸盐形式，这是进一步将吉西他滨转变为有活性的三磷酸核苷的限速步骤，同时对于吉西他滨的活性尤为重要。Dck作为吉西他滨转化为活性形式的限速酶，其表达水平与吉西他滨药物敏感性有着一定的关系。

荷兰VU大学医学中心Kroep等^[9]^研究了包括肺癌在内的8种不同起源的小鼠移植瘤组织的dck表达与吉西他滨敏感性关系，结果显示dck活性以及其蛋白表达水平与吉西他滨敏感性呈正相关（*P* < 0.001），同时dck mRNA表达水平与dck活性以及蛋白表达水平明显相关，但是并未发现dck mRNA表达水平与吉西他滨敏感性相关，可能与样本含量太小有关，尚需要进一步研究dck mRNA表达水平与吉西他滨敏感性的关系。日本名古屋大学Achiwa等^[3]^指出dck mRNA表达水平下调与NSCLC吉西他滨获得性耐药有关，但是在22种NSCLC细胞株中未发现dck mRNA表达水平与吉西他滨敏感性明显相关。法国一项小样本临床回顾性研究^[10]^采用免疫组化方法研究了43例均接受含吉西他滨方案化疗的NSCLC患者的肿瘤组织的dck蛋白表达水平与患者临床预后的关系，其中28例dck蛋白表达阳性，但是未发现其表达水平与临床预后有明显相关性。

### 2.2 cN-II 5’-NT（nucleotidase）

cN-II 5’-NT为细胞内重要的核苷酸去磷酸化酶，吉西他滨的磷酸化代谢产物可能被该酶降解，因此其表达水平可能与吉西他滨药物敏感性有一定关系。法国Seve等^[10]^同时还研究了43例接受含吉西他滨化疗的NSCLC患者cN-II蛋白表达水平与临床预后的关系，结果显示cN-II蛋白低表达者（阳性染色细胞 < 40%）的总生存期明显比高表达者短，分别为6个月、11个月（*P*=0.047）；同时对年龄、性别、组织类型、分期、体重以及免疫组化结果进行*COX*多因素变量分析，结果显示，cN-II表达水平可以作为总生存期的独立预后因素（*P*=0.02）。

### 2.3 胞苷脱氨酶（cytidine deaminase, CDA）

吉西他滨也可以被CDA脱氨基引起其失活，Eliopoulos以及Neff等^[11, 12]^研究认为转染细胞中的CDA过表达降低了吉西他滨的敏感性。并且有研究^[13]^表明，许多CDA启动子区域的单核苷酸多态性（single nucleotide polymorphism, SNP）可能影响其表达以及活性。意大利Tibaldi等^[14]^报道，在接受顺铂/吉西他滨方案化疗的进展期NSCLC高加索人种患者中，CDA第27位密码子基因多态性与其疗效明显相关。携带CDA27 Lys/Lys的患者中位疾病进展时间（8.4个月）及中位总生存期（13.6个月）比携带CDA27 Lys/Gln（5.0个月、13.6个月）以及CDA27 Gln/Gln（3.3个月、4.1个月）基因型患者明显延长（*P*=0.006, *P*=0.002）；与携带Lys/Gln、Gln/Gln基因型患者相比，携带Lys/Lys的患者临床获益率明显提（92.6% *vs* 75.9%, 55.6%; *P*=0.04），同时3级及以上中性粒细胞、血小板减少毒副作用增加，其机制可能与携带CDA27 Lys/Lys基因型患者CDA活性较低有关。新加坡国立大学Soo等^[15]^的研究表明，*CDA*基因第435位密码子的多态性与临床也明显相关。在接受含吉西他滨方案化疗的53例晚期NSCLC亚裔患者中，携带CDA453 C/C基因型患者的中位疾病进展时间明显长于携带CDA453 C/T及T/T基因型的患者（6.3个月*vs* 3.3个月，*P*=0.018），且客观缓解率明显提高（62% *vs* 29%, *P*=0.017）。

## 3 药物作用靶点相关基因

### 3.1 核糖核苷酸还原酶（ribonucleotide reductase, RR）

RR是DNA合成通路中的限速酶，它能使核苷酸转化为脱氧核苷酸，后者是DNA合成和修复中必不可少的原料。RR包括2个亚单位：核糖核苷酸还原酶亚基1（ribonucleotide reductase subunit1, RRM1）和RRM2。RRM1是核苷酸结合位点，控制底物的特异性和整个酶的活性，同时也是核苷类似物系的化疗药物的结合位点；RRM2是一个金属结合位点，有携带接触抑制区，控制底物转化的功能^[16]^。吉西他滨二磷酸盐可抑制RR，减少DNA合成和修复所需的脱氧核苷酸量，尤其是三磷酸脱氧核苷，使DNA合成障碍。Davidson等^[17]^研究认为RRM1可能是吉西他滨抗肿瘤作用的主要靶点。

Davidson等^[17]^选择NSCLC细胞株H358和H460进行的一项体外实验发现，吉西他滨耐药细胞株中的RRM1 mRNA表达水平以及蛋白表达水平明显高于对照组（吉西他滨敏感组），因此认为RRM1表达水平和吉西他滨敏感性有关。H. Lee Moffitt癌症研究中心Bepler等^[18]^选择H23细胞株进行体外研究了RRM1 mRNA表达水平与吉西他滨IC_50_关系，H23-R1（吉西他滨耐药株）RRM1 mRNA表达水平比对照组高2.5倍-3.5倍，其吉西他滨IC_50_值比对照组高8倍，而H23siR1（吉西他滨敏感株）RRM1 mRNA表达水平比对照组低5倍，其吉西他滨IC_50_值比对照组低10倍，因此，可以认为RRM1 mRNA高表达可导致吉西他滨耐药，而低表达增加吉西他滨的敏感性。

一项来自H. Lee Moffitt癌症研究中心的大样本临床回顾性研究^[19]^显示，187例仅接受手术切除的Ⅰ期NSCLC患者中，肿瘤组织RRM1 mRNA高表达者总生存期（> 120个月*vs* 60.2个月，*P*=0.02）、无疾病生存期（120个月*vs* 60.2个月，*P*=0.004）均明显长于低表达者，因此RRM1 mRNA表达水平是生存预后指标。而国内外多项临床回顾性研究发现，在晚期接受化疗的NSCLC患者RRM1 mRNA低表达提示预后较好。西班牙肺癌协作组织Rosell等^[20]^采用RT-PCR方法对100例晚期NSCLC患者的石蜡组织进行检测，发现21例接受吉西他滨/顺铂治疗患者中RRM1 mRNA低表达组较高表达组的中位生存期、无疾病进展期均明显延长（13.7个月*vs* 3.6个月，*P*=0.009；8.4个月*vs* 2.7个月，*P*=0.020）；该小组织成员^[21]^又对67例Ⅱb期、Ⅲa期和Ⅲb期术后接受含吉西他滨方案化疗的NSCLC患者肿瘤组织进行检测，进一步证实了RRM1 mRNA低表达组较高表达组的死亡风险降低了70%，中位生存期明显延长（52个月*vs* 26个月，*P*=0.018），并且RRM1 mRNA低表达组具有更好的肿瘤缓解率、完全切除率和病理完全缓解率。Ceppi等^[22]^对70例晚期NSCLC患者进行的回顾性研究，也证实RRM1 mRNA低表达组中位生存期明显延长（13.9个月*vs* 10.9个月，*P*=0.039）。中南大学湘雅医院梁伟军等^[23]^采用免疫组化方法研究了RRM1蛋白表达水平与NSCLC术后吉西他滨联合顺铂辅助化疗预后的关系，发现RRM1蛋白低表达组中化疗组和未化疗组中位生存期分别为42个月和22个月（*P*=0.010），RRM1高表达组中化疗组和未化疗组中位生存期分别为28个月和21个月（*P*=0.092），可以看出RRM1蛋白低表达的NSCLC患者更能从术后含吉西他滨顺铂方案化疗中获益，与国外研究结果一致。

H. Lee Moffitt癌症研究中心Bepler等^[18]^在对35例局部进展期NSCLC患者设计的一项前瞻性的Ⅱ期临床试验中，采用RT-PCR技术检测RRM1 mRNA表达水平，发现完成了2个周期吉西他滨/顺铂方案化疗患者的RRM1 mRNA表达水平与临床疗效呈负相关（*r*=-0.498, *P*=0.002），因此认为接受吉西他滨/顺铂方案化疗患者其肿瘤组织RRM1 mRNA低表达者更能获益。该中心2009年进一步完成的Ⅲ期临床试验^[24]^也同样证实了上述结论。

RRM1多个位点的单核苷酸多态性与接受吉西他滨化疗的NSCLC患者预后亦有一定的关系。有研究^[25]^表明，RRM1第2, 464位密码子多态性与吉西他滨敏感性有关，在62种肿瘤细胞中与携带野生型RRM12464G/G相比，携带RRM12464G/A基因型细胞对吉西他滨更加敏感（*P*=0.011）。Bepler等^[26]^对286例术后白种及非洲美裔人群NSCLC患者RR第37位及第524位密码子联合分析，结果显示携带RR37CC-RR524TT基因型患者总生存期及无疾病生存期更长。但广东肺癌协会一项亚裔人种*RRM1*基因多态性研究^[27]^提示携带RRM1C(-)37A患者无进展生存期较携带A/A、C/C基因型患者明显延长（30.7周*vs* 24.7周*vs* 23.3周，*P*=0.043），携带RRM1C(-)37A患者更能从含吉西他滨化疗中获益。而北京军事医学科学院附属医院林莉等报道^[28]^，110例肺癌患者血标本中RRM1(-)37位点的基因型与肺癌患者的一般状态，疗效及生存时间均无相关性。

### 3.2 DNA修复相关基因

众所周知，DNA修复能力是影响铂类药物疗效的重要原因，而铂类药物联合吉西他滨是临床上NSCLC最常用的化疗方案之一，同时由于吉西他滨的主要作用机制是影响DNA的合成和修复，因此DNA修复能力亦可能是影响吉西他滨药物抵抗的重要原因之一。DNA切除修复主要有碱基切除修复（base-excision repair, BER）、DNA双链断裂修复（DNA double-strandbreak repair, DDSBR）、错配修复（mismatch repair, MMR）及核苷酸切除修复（nucleotide- excision repair, NER）4种途径^[29]^。

#### 3.2.1 切除修复交叉互补基因1（excision repair crosscomplementary 1, ERCC1）

*ERCC1*是NER中的重要基因，在DNA修复途径中起了关键性作用，而吉西他滨主要作用靶点之一RRM1，可以提供脱氧核苷酸，用于填补由于ERCC1切除所产生的空隙，从而参与DNA链修复^[30]^。许多临床研究^[20, 24]^表明，ERCC1 mRNA或蛋白表达水平与接受含吉西他滨方案化疗的NSCLC患者的预后有关。Rosell等^[20]^采用RT-PCR方法对100例晚期NSCLC患者的石蜡组织进行联合基因检测，结果表明ERCC1 mRNA与RRM1 mRNA表达水平明显相关（*r*=0.410, *P* < 0.001)，在21例接受吉西他滨/顺铂治疗的患者中，ERCC1 mRNA与RRM1 mRNA均低表达者较两者均高表达患者中位生存期明显延长（*P*=0.016）。H. Lee Moffitt癌症研究中心2009年完成的一项前瞻性Ⅲ期临床试验^[24]^也得出相同的结论：170例NSCLC患者接受吉西他滨单药或卡铂/吉西他滨治疗后，原位RRM1、ERCC1蛋白表达水平与疾病缓解呈明显负相关（*r*=-0.41, *P*=0.001; *r*=-0.39, *P*=0.003）。有体外研究^[31]^显示：*ERCCI*基因第118位密码子的基因多态性与其mRNA及蛋白表达水平相关。韩国Ryu等^[32]^对亚裔人群进行的研究证实了接受吉西他滨/顺铂方案化疗的进展期NSCLC患者携带ERCC1 codon118 C/C基因型总生存期明显较C/T及T/T基因型患者延长（*P*=0.005, 8）；但是Tibaldi、Zhou等^[14, 33]^通过对中晚期NSCLC患者进行研究，提出一个相反的结果，即ERCC1 codon118 C/C基因型生存期较C/T、T/T型明显减少，但未达到统计学差异（*P*=0.41, *P*=0.37）。

#### 3.2.2 着色性干皮病基因D（xeroderma pigmentosum pomplementary group D, XPD）

XPD又称作ERCC2，在NER系统中作为进化保守的DNA解旋酶，发挥5’→3’解旋酶活性，参与两种NER途径和基因转录^[34]^。*XPD*的基因多态性可以改变DNA修复能力。Rosell等^[35]^研究表明，*XPD*基因第751位密码子SNP与化疗效果的关系与化疗药物的联合使用有关，在接受吉西他滨+顺铂治疗的进展期NSCLC患者中，具有A/C基因型患者的疾病进展时间明显长于具有A/A基因型的患者（9.6个月*vs* 4.2个月，*P*=0.03）。

#### 3.2.3 X线修复交叉互补基因3（X-ray cross-complementing group3, XRCC3）

XRCC3是参与DNA的同源重组修复途径从而保持染色体的稳定性和修复DNA损伤重要基因之一。XRCC3编码的蛋白在同源重组修复过程中起着重要作用，其第7外显子C→T变异，导致241位密码子Thr→Met，该密码子的多态性和吉西他滨的敏感性有一定关系。西班牙肺癌协作组^[36]^采用探针法检测135例接受吉西他滨/顺铂化疗的Ⅲb或Ⅳ期肺癌患者外周血中*XRCC3*基因的多态性进行分析，发现XRCC3第241密码子基因型与患者的生存期明显相关，Met/Met型患者中位生存时间为16个月，Thr/Thr型为14个月，Thr/Met型仅为10个月（*P*=0.01）。

#### 3.2.4 乳腺癌易感基因1（breast cancer susceptibility gene 1, BRCA1）

BRCA1通过参与细胞内DNA修复、细胞周期调节、mRNA转录调控及蛋白捕获等途径而对DNA损害应答^[37]^，同时还被证实有调节有丝分裂的作用，与具有纺锤体毒性的药物（紫杉类、长春碱类）敏感性有关^[38]^。Taron等^[39]^研究小组报道，对55例手术切除并接受吉西他滨/顺铂新辅助化疗的Ⅱb期-Ⅲb期的NSCLC标本检测BRCA1 mRNA表达水平，结果BRCA1 mRNA高表达的患者中位生存期为12.7个月，中表达的28例患者中位生存期为37.8个月，低表达的15例患者尚未达到中位生存时间；而Boukovinas等^[40]^报道，对102例接受吉西他滨/多西他赛一线治疗的进展期NSCLC患者的石蜡组织进行*BRCA1*、*RRM1*、*RRM2*联合基因检测，发现BRCA1 mRNA与RRM1 mRNA表达水平正相关（*r*=0.27, *P*=0.008），与RRM2 mRNA表达水平呈负相关（*r*=-0.25, *P*=0.02），且随着BRCA1 mRNA表达水平增高，其临床疗效增加[优势比（odds ratio, OR）=1.09，*P*=0.01]，疾病进展风险下降[危害比（hazard ratio, HR）=0.99，*P*=0.36]；而随着RRM1 mRNA和RRM2 mRNA表达水平增高，临床疗效（RRM1: OR=0.97, *P*=0.82; RRM2: OR=0.94, *P*=0.000, 1)下降，疾病进展风险（RRM1: HR=1.02, *P*=0.001; RRM2: HR=1.005, *P*=0.01）增高。同时对RRM1 mRNA和BRCA1 mRNA表达水平联合分析显示，低危、中危、高危三组疾病进展时间（times to progression, TTP）分别为10.13个月、4.17个月、2.30个月（*P*=0.001）。因此，我们可以推论：BRCA1 mRNA低表达的NSCLC患者可以从吉西他滨/顺铂联合方案化疗中获益，而高表达者则可以从多西他赛联合吉西他滨化疗中获益。

#### 3.2.5 ATM/chek2及ATR/chk1信号转导通路相关基因

ATM和ATR属于磷脂酰肌醇-3-激酶样激酶家族，是DNA损伤检查点的主要成员。它们被不同类型的DNA损伤所激活，通过磷酸化相应的下游蛋白Chk1和Chk2等，调节细胞周期各个检查点，引起细胞周期阻滞，使DNA损伤得以修复。多项体外研究^[41, 42]^显示：吉西他滨引起的细胞DNA复制受损可以激活这两条信号传导通路，而这两条信号转导通路相关蛋白如ATM、chk1、ATR等表达缺失可以增加吉西他滨对包括NSCLC在内的多种肿瘤细胞的药物敏感性，但是尚缺乏大量回顾性及前瞻性临床试验的验证。

因此，*ERCC1*、*XPD*、*XRCC3*、*BRCA1*等DNA修复相关基因可以作为NSCLC患者以吉西他滨为基础的个体化治疗的潜在临床预测指标，但是仍需要大量的多中心大样本临床前瞻性研究。而ATM/Chek2及ATR/Chk1信号转导通路相关基因与吉西他滨敏感性的相关性只在一些体外实验中得以证实，尚缺乏临床试验的验证。

### 3.3 细胞凋亡相关基因

吉西他滨部分通过诱导细胞凋亡而发挥其细胞毒作用，因此与细胞凋亡相关的信号转导通路的相关基因或蛋白的表达可能与吉西他滨药物抵抗相关。*p53*是一原癌基因，在细胞凋亡、基因组的稳定性、细胞衰老以及分化、生长停滞方面起重要作用，该基因的突变常导致其功能缺失，是人类肿瘤最常见的一种基因改变。但是由于*p53*基因检测方法的多样性以及其特殊的方法步骤、评价标准，使得其是否突变的状态与包括吉西他滨等抗肿瘤药物敏感性及临床预后的关系上尚存在很大争议。一项体外研究认为^[43]^，吉西他滨是通过依赖Bcl-2的激活半胱天冬酶9的途径诱导NSCLC细胞凋亡的，与*p53*是否突变无明显相关性，其主要是通过激活胞外信号调节激酶（extracellular signal-regulated kinase, ERK）、下调bcl-2以及AKT失活而引起细胞凋亡。而一项基于4, 571例NSCLC患者的回顾性研究^[44]^却发现：用免疫组化方法检测肿瘤组织的p53蛋白表达情况，其中p53蛋白表达阳性者对吉西他滨更耐药（*P*=0.010, 8）。另外，韩国首尔大学Lee等^[45]^报道：通过测序法检测57例接受吉西他滨作为一线或二线化疗的NSCLC患者的*EGFR*、*K-ras*突变状态及用免疫组化方法检测患者石蜡包埋肿瘤组织的p-Erk、p-Akt蛋白表达水平，结果显示：EGFR、K-ras状态以及p-Akt蛋白表达水平与患者的疾病进展时间、疾病客观缓解率均无明显相关性，而p-Erk(+)患者客观缓解率明显高于阴性患者（30.6% *vs* 0%, *P*=0.01）。

*PNAS-4*是在大规模测序中确定的一个新的基因，它的过表达可以增加细胞凋亡^[46]^。Hou^[47]^等研究发现，在A459肺癌细胞株体外药敏试验中，PNAS-4高表达组细胞活性比对照组低18%；吉西他滨处理后，PNAS-4高表达组与对照组相比，细胞活性明显降低（36% *vs* 70%）；流式细胞仪测定细胞凋亡实验显示，PNAS-4高表达/吉西他滨组凋亡比例明显比单独吉西他滨处理组高（61.4% *vs* 28.6%）；且体内研究表明PNAS-4可以抑制A459小鼠移植瘤的生长，与PNAS-4、吉西他滨单独使用相比，两者联合应用可以增加肿瘤组织内细胞凋亡，但是并不引起更大的毒副作用，因此，PNAS-4可以抑制肿瘤生长并且通过凋亡作用增加吉西他滨的敏感性。*PNAS-4*基因有可能作为治疗肺癌的潜力候选者。

## 4 小结及展望

吉西他滨是目前NSCLC常用化疗药物之一，其药物抵抗是个复杂的过程，有多种基因参与，通过对患者相关基因或蛋白表达水平以及基因遗传多态性的分析，可以指导临床选择最佳治疗方案，从而提高药物治疗的有效率，避免严重毒副反应发生，延长患者的生存期，提高生活质量，真正实现个体化治疗。

吉西他滨首先通过hENT1等药物转运相关载体至细胞内，通过抑制RR及DNA合成从而发挥其抗肿瘤作用。dck是吉西他滨转化为其活性形式从而发挥其抗肿瘤作用的限速酶，而5’-NT、CDA则是主要的导致其失活的酶。从上述关于NSCLC吉西他滨药物耐药相关基因研究的文章中可以看出：（1）药物转运相关基因：hENT1 mRNA高表达或蛋白表达阳性提示吉西他滨敏感，可获得更好的生存优势，但目前在NSCLC方面，国外研究主要对象集中于体外细胞株，临床回顾性研究较少，且尚未出现前瞻性研究，国内关于hENT1则罕见报道；（2）药物代谢相关基因：体外研究显示dck活性以及蛋白表达水平与吉西他滨敏感性呈正相关，但是并未发现dck mRNA表达水平与吉西他滨敏感性明显相关，在极少的临床回顾性研究中并未发现阳性结果；关于CDA的研究主要集中于单核苷酸多态性与临床预后的关系研究，携带CDA27Lys/Lys、CDA453C/C基因型患者更能从含吉西他滨化疗方案中获益，获得生存优势；而在含吉西他滨化疗的NSCLC患者中，cN-II蛋白低表达者的总生存期明显比高表达者短；（3）药物作用靶点相关基因：关于RRM1 mRNA表达水平以及其基因多态性与吉西他滨药物敏感性和接受吉西他滨化疗的NSCLC患者临床预后关系，国内外报道很多。从大量体外研究及临床研究中可以发现，RRM1 mRNA表达水平可以作为吉西他滨敏感性及其临床疗效预测的重要分子标志。在中晚期接受化疗的NSCLC患者中RRM1 mRNA高表达提示预后较差，而早期仅接受手术治疗患者RRM1高表达者预后较好。同时，由于种族差异性，RRM1的多个位点的单核苷酸多态性与RRM1 mRNA表达水平间尚没有发现有一定的联系，且相同位点多核苷酸多态性与临床预后的关系并不一致；由于吉西他滨主要通过影响DNA合成和修复，从而抑制肿瘤细胞分裂，诱导肿瘤细胞凋亡发挥细胞毒作用，因此DNA修复相关基因，如*ERCC1*、*BRCA1*、*XRCC3*、*XPD*等基因表达水平或其单核苷酸多态性亦与接受含吉西他滨方案化疗的NSCLC患者预后有一定的相关性，但由于种族差异，各临床研究得出的结论不尽相同；而细胞凋亡相关基因与吉西他滨药物敏感性相关关系方面仍存在很大争议，需要进一步的研究加以证实。

目前在国内外开展的关于NSCLC吉西他滨药物敏感性基因的体内外研究中，部分基因研究结果并不一致，选择合适的分子预后指标还需要大量的大样本临床回顾性及多中心临床前瞻性研究；在基因分子选择上多限于单个基因的表达谱或多态性研究，联合基因检测报道并不多，显然，多基因的全面分析可能更具有临床应用潜力；相关基因表达水平的检测标本主要是手术切除或者支气管镜活检获得的肿瘤组织，相对难以获取，如何通过其他临床易得样本如外周血标本等进行基因检测，也是一个尚待解决的问题。同时，我们还需要进一步进行分子机制等基础研究以寻找到更多的与吉西他滨药物耐药相关的基因，从而进一步指导药物的临床应用。
